# Melittin-Based Nanoparticles for Cancer Therapy: Mechanisms, Applications, and Future Perspectives

**DOI:** 10.3390/pharmaceutics17081019

**Published:** 2025-08-06

**Authors:** Joe Rizkallah, Nicole Charbel, Abdallah Yassine, Amal El Masri, Chris Raffoul, Omar El Sardouk, Malak Ghezzawi, Therese Abou Nasr, Firas Kreidieh

**Affiliations:** 1 Department of Diagnostic Radiology, American University of Beirut, Beirut P.O. Box 11-0236, Lebanon; jr56@aub.edu.lb (J.R.); oe34@aub.edu.lb (O.E.S.); 2 Division of Hematology and Oncology, Department of Internal Medicine, American University of Beirut, Beirut P.O. Box 11-0236, Lebanon; nc47@aub.edu.lb (N.C.); ay53@aub.edu.lb (A.Y.); axm09@mail.aub.edu (A.E.M.); cgr04@mail.aub.edu (C.R.); 3 Department of Surgery, American University of Beirut, Beirut P.O. Box 11-0236, Lebanon; mg96@aub.edu.lb; 4 Chronic Care Center, Hazmieh P.O. Box 213, Lebanon; nasrnabhan@doctor.com

**Keywords:** melittin, cancer therapy, nanomedicine, tumor targeting, combination therapy, tumor microenvironment

## Abstract

Melittin, a cytolytic peptide derived from honeybee venom, has demonstrated potent anticancer activity through mechanisms such as membrane disruption, apoptosis induction, and modulation of key signaling pathways. Melittin exerts its anticancer activity by interacting with key molecular targets, including downregulation of the PI3K/Akt and NF-κB signaling pathways, and by inducing mitochondrial apoptosis through reactive oxygen species generation and cytochrome c release. However, its clinical application is hindered by its systemic and hemolytic toxicity, rapid degradation in plasma, poor pharmacokinetics, and immunogenicity, necessitating the development of targeted delivery strategies to enable safe and effective treatment. Nanoparticle-based delivery systems have emerged as a promising strategy for overcoming these challenges, offering improved tumor targeting, reduced off-target effects, and enhanced stability. This review provides a comprehensive overview of the mechanisms through which melittin exerts its anticancer effects and evaluates the development of various melittin-loaded nanocarriers, including liposomes, polymeric nanoparticles, dendrimers, micelles, and inorganic systems. It also summarizes the preclinical evidence for melittin nanotherapy across a wide range of cancer types, highlighting both its cytotoxic and immunomodulatory effects. The potential of melittin nanoparticles to overcome multidrug resistance and synergize with chemotherapy, immunotherapy, photothermal therapy, and radiotherapy is discussed. Despite promising in vitro and in vivo findings, its clinical translation remains limited. Key barriers include toxicity, manufacturing scalability, regulatory approval, and the need for more extensive in vivo validation. A key future direction is the application of computational tools, such as physiologically based pharmacokinetic modeling and artificial-intelligence-based modeling, to streamline development and guide its clinical translation. Addressing these challenges through focused research and interdisciplinary collaboration will be essential to realizing the full therapeutic potential of melittin-based nanomedicines in oncology. Overall, this review synthesizes the findings from over 100 peer-reviewed studies published between 2008 and 2025, providing an up-to-date assessment of melittin-based nanomedicine strategies across diverse cancer types.

## 1. Introduction

Cancer remains one of the most significant global health challenges of the 21st century. According to the World Health Organization, cancer accounted for nearly 10 million deaths in 2020, making it the second leading cause of mortality worldwide [1]. The global incidence of cancer is projected to rise from 19.3 million new cases in 2020 to 28.4 million by 2040, largely driven by population aging and shifting lifestyle patterns [1]. Although substantial progress has been made in early detection, surgery, chemotherapy, targeted therapy, and immunotherapy, several limitations persist. These include drug resistance, systemic toxicity, and poor selectivity between malignant and healthy cells [2]. To address these challenges, researchers have prioritized the development of novel therapeutic agents capable of selectively eradicating tumor cells while minimizing the harm to normal tissues. Among emerging strategies, peptide-based therapies—particularly those derived from natural sources such as melittin—have attracted growing interest due to their potential to overcome these barriers [3,4].

**Figure 1:**
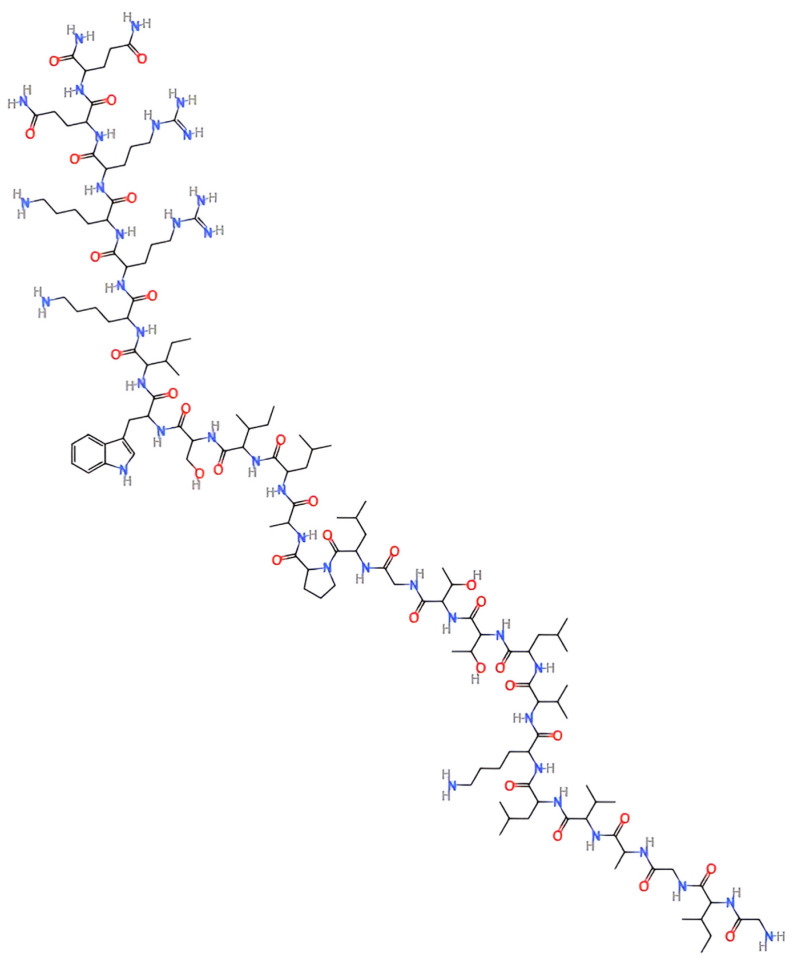
Two-dimensional chemical structure of melittin peptide.

Melittin is a 26-amino-acid, cationic, amphipathic peptide that constitutes approximately 40–50% of the dry weight of *Apis mellifera* (honeybee) venom (Figure 1). It has been extensively studied for its broad spectrum of biological activities, including its anti-inflammatory, antimicrobial, antiviral, and, notably, anticancer effects. Structurally, melittin contains a hydrophobic N-terminal region and a hydrophilic C-terminal region, which enable it to adopt an α-helical conformation in lipid environments. This amphipathic nature allows melittin to intercalate into lipid bilayers, where it forms pores or disrupts membrane integrity, ultimately causing cell lysis [5,6]. In cancer models, melittin exhibits multiple anticancer mechanisms. It disrupts cancer cell membranes through direct cytolysis, resulting in rapid cell death [6,7]. It also induces apoptosis by permeabilizing the mitochondrial membrane, which leads to the production of reactive oxygen species (ROS) [8]. Furthermore, melittin suppresses tumor progression by modulating key signaling pathways involved in cell survival and proliferation, including the nuclear factor kappa B (NF-κB), phosphoinositide 3-kinase/protein kinase B (PI3K/Akt), and mitogen-activated protein kinase (MAPK) pathways [7].

Although the broad cytotoxicity of melittin facilitates cancer cell destruction, it poses major challenges for therapeutic application due to its limited selectivity and high systemic toxicity [7,9]. Melittin lyses a wide range of cell types, including red blood cells, resulting in hemolysis—a significant barrier to systemic administration [9]. It is also rapidly degraded in blood and plasma; demonstrates poor pharmacokinetics and limited in vivo stability; and can provoke allergic immune responses because of its immunogenic nature [6,9]. These limitations have hindered its clinical translation, despite the strong anticancer activity observed in vitro, highlighting the need for targeted and protective delivery systems to enable safe and effective treatment [4,7,9].

To address these limitations, nanotechnology-based delivery systems have been investigated for enhancing the therapeutic potential of melittin [7,9]. A range of nanocarriers—including liposomes, polymeric nanoparticles, micelles, and metal-based nanoparticles—have been designed to improve tumor specificity, reduce systemic toxicity, and protect melittin from degradation. In addition, stimuli-responsive platforms offer controlled release within the tumor microenvironment (TME) [10]. Although most of this progress remains in the preclinical phase, these strategies show considerable promise in improving the safety and efficacy of melittin for cancer treatment [9,10].

This review provides an updated synthesis of melittin as a therapeutic agent in oncology and critically evaluates the application of nanotechnology-based delivery systems to overcoming the key challenges to its clinical use, particularly toxicity and poor specificity. Although previous reviews have addressed selected aspects of melittin’s anticancer potential—such as its mechanisms, delivery platforms, and immunomodulatory effects—this review offers a uniquely comprehensive and up-to-date synthesis. It integrates the mechanistic insights and preclinical findings across 18 cancer types while critically examining persistent translational barriers such as systemic toxicity, manufacturing scalability, and regulatory limitations. To our knowledge, this is the first and only review to address melittin-based nanomedicine across such a wide range of cancer types. Beyond summarizing the existing literature, this work adds value through a cross-cutting analysis that compares the melittin-based strategies across diverse delivery systems and cancer contexts, identifies the patterns in its preclinical efficacy, and proposes future directions grounded in emerging technologies. By doing so, this review aims to support the development of clinically viable melittin-based nanomedicine and provide a roadmap for translational research.

## 2. Mechanisms of Action of Melittin in Cancer Therapy

Melittin, the principal active component of bee venom, has shown significant anticancer activity across a range of malignancies, including prostate, ovarian, lung, breast, hepatocellular, and cervical cancers, as well as melanoma and several others. Traditionally used in Chinese medicine to manage inflammatory and autoimmune disorders such as rheumatoid arthritis, bee venom—specifically melittin—has been increasingly investigated for its role in tumor suppression through multiple molecular mechanisms [11].

### 2.1. Its Effect on the Cell Cycle

An important effect of melittin in cancer cells is the induction of apoptosis. In gastric cancer models, melittin has been shown to initiate mitochondrial-dependent apoptosis, characterized by increased generation of ROS, disruption of the mitochondrial membrane potential, and the release of apoptotic factors such as cytochrome c and endonuclease G. These events lead to caspase-3 activation and morphological changes in the treated cells, culminating in programmed cell death [12]. In cervical cancer, melittin extracted from Iranian honeybee venom inhibited HeLa cell proliferation and induced apoptosis in a dose-dependent manner, as demonstrated through the 3-(4,5-dimethylthiazol-2-yl)-2,5-diphenyltetrazolium bromide (MTT) assay and the expression of apoptotic markers [13]. Supporting this, Kim et al. reported that bee venom suppressed cervical cancer cell proliferation and migration by downregulating human papillomavirus E6/E7 expression [14]. Similar apoptotic activity has also been observed in colorectal cancer (CRC) cell lines, where melittin modulated key regulatory proteins by upregulating Bax, downregulating Bcl-2, and activating the caspase cascade [15].

Melittin has also demonstrated the ability to induce cell cycle arrest in various cancer models. In liver cancer, melittin was shown to suppress Rac1-dependent signaling pathways, resulting in microfilament depolymerization and reduced motility in HepG2 human hepatic cells in a dose- and time-dependent manner. Notably, these effects were accompanied by the increased expression of the tumor suppressor p53 and cytochrome c, along with the downregulation of cyclin D1, collectively leading to growth arrest [16].

### 2.2. Modulating Signaling Cascades

Melittin also contributes to anticancer activity by modulating several critical signaling pathways. In non-small-cell lung cancer (NSCLC), Yu et al. reported that melittin downregulated the transforming growth factor beta-mediated extracellular signal-regulated kinase pathway, thereby inhibiting cancer cell proliferation, migration, and invasion [17]. In breast cancer, melittin suppressed the activation of human epidermal growth factor receptor 2 (HER2) and epidermal growth factor receptor (EGFR), two major drivers of tumor progression in HER2-enriched and triple-negative subtypes [18].

Melittin also inhibited epidermal growth factor (EGF)-induced cell motility and suppressed the expression of matrix metalloproteinase (MMP)-9 by blocking the NF-κB and PI3K/Akt/mammalian target of rapamycin (mTOR) signaling axes [19]. Additional studies have shown that melittin downregulates oncogenic pathways, including Akt, NF-κB, hypoxia-inducible factor 1-alpha (HIF-1α), Wnt, and signal transducer and activator of transcription 3 (STAT3), further supporting its ability to interfere with cancer-associated signaling networks [20,21].

Melittin has demonstrated strong antimetastatic activity by reducing tumor cell invasion and migration. In gastrointestinal cancers—particularly in AGS gastric cancer cells—melittin decreased cellular motility and inhibited the epithelial–mesenchymal transition (EMT) by modulating the Wnt/bone morphogenetic protein (BMP) signaling pathway. It also downregulated several metastasis-associated markers, including MMP-2, MMP-9, vascular endothelial growth factor (VEGF), and tumor necrosis factor (TNF) [22].

### 2.3. Chemotherapy and Radiotherapy Sensitization

Melittin also shows promise as a sensitizing agent in both chemotherapy and radiotherapy. In breast cancer mouse models, the combination of melittin and irradiation significantly reduced the clonogenic potential and tumor volume, with the decreases in tumor cell growth rates ranging from 18% to 84% across different breast cancer cell lines and melittin concentrations, while increasing the Bax/Bcl-2 ratio 1.62-fold in 4T1 cells and 4.82-fold in MCF-7 cells, indicating enhanced apoptotic sensitivity [23]. Furthermore, in a study by Yan et al. on hypoxic head and neck squamous cell carcinoma (HNSCC), melittin treatment downregulated VEGF and HIF-1α, both commonly upregulated in radioresistant tumors. In this model, combination therapy extended the tumor doubling time from 7.08 to 10.06 days (compared to that under irradiation alone) and reduced the surviving fraction in hypoxic HNSCC cells from 0.86 to 0.69, confirming its synergistic radiosensitizing effect. These findings suggest that melittin enhances radiosensitivity by targeting hypoxia-related mechanisms [24].

**Figure 2:**
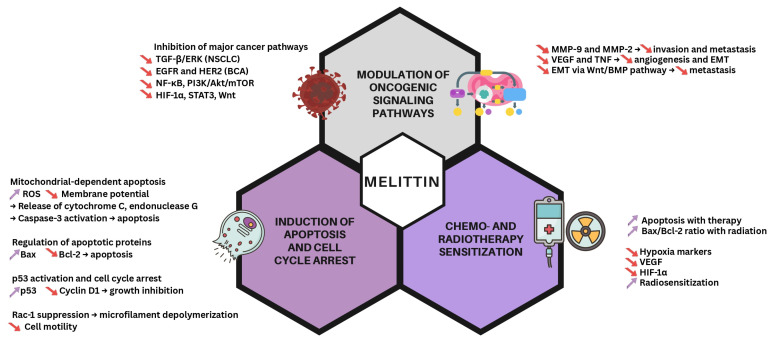
Mechanisms of action of melittin in cancer therapy. Downward arrows indicate a decrease in the corresponding variable, whereas upward arrows indicate an increase.

These multifaceted mechanisms—including modulation of signaling pathways, induction of apoptosis, and sensitization to chemo- and radiotherapy—are summarized in Figure 2.

## 3. Melittin-Based Nanoparticles: From the Shelf to the Bedside

### 3.1. Delivery Systems

**Figure 3:**
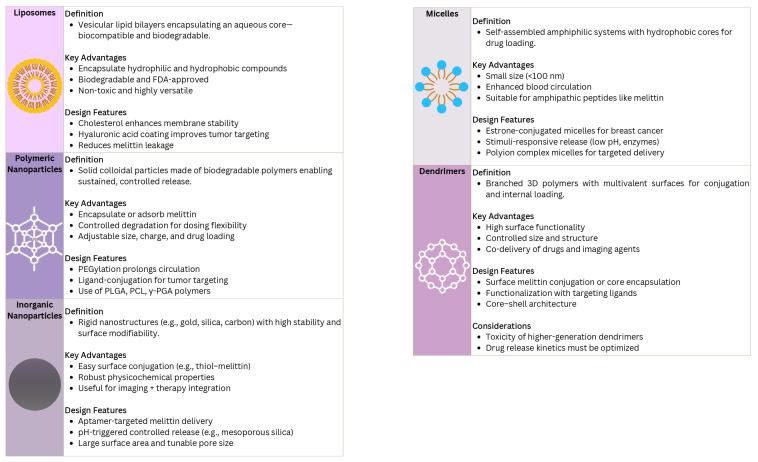
An overview of melittin nanocarrier systems and their design characteristics.

The effective clinical translation of melittin as an anticancer agent depends on advanced delivery systems that can reduce its systemic toxicity while maximizing the concentration at the tumor site [10]. Nanotechnology provides a wide range of platforms for achieving this goal, often classified by their composition and structure (Figure 3). These systems can enhance stability, bioavailability, and tumor targeting while also helping to overcome mechanisms of drug resistance [10,25]. Liposomes—vesicular structures consisting of lipid bilayers surrounding an aqueous core—are among the most extensively studied nanocarriers for drug delivery, including for peptides such as melittin [26]. Their biocompatibility, biodegradability, and ability to encapsulate both hydrophilic and hydrophobic compounds make them especially appealing. Structural modifications, such as the incorporation of cholesterol or surface coating with materials like hyaluronic acid, can further improve liposome stability, reduce melittin leakage, and enhance tumor-specific delivery [26].

Polymeric nanoparticles, formed from biodegradable and biocompatible polymers such as poly(lactic-co-glycolic acid), poly(caprolactone), or poly-γ-glutamic acid, represent another versatile strategy for melittin delivery [27,28]. These systems can encapsulate melittin within the polymer matrix or adsorb it onto the particles’ surface, enabling controlled and sustained release profiles [27]. The polymer degradation rate can be adjusted to align with the intended therapeutic window, potentially reducing the dosing frequency. Furthermore, the surface chemistry of polymeric nanoparticles allows for functionalization with targeting ligands or stealth coatings such as polyethylene glycol (PEG), which can prolong the circulation time and improve the tumor specificity [10,27]. The ability to fine-tune the particle size, surface charge, and drug loading capacity makes polymeric nanoparticles a highly adaptable platform for delivering melittin in diverse cancer models [27].

Dendrimers, defined by a highly branched, three-dimensional architecture extending from a central core, offer distinct advantages for peptide delivery due to their multivalency and controlled structure [29,30]. This well-organized architecture enables precise control over size, shape, and surface functionality, making dendrimers suitable candidates as nanoscale delivery vectors [29,30]. Melittin can either be conjugated to surface functional groups or encapsulated within the internal cavities of dendrimers. A high density of surface groups supports the attachment of multiple melittin molecules, along with targeting ligands or imaging agents [10,29]. Despite their potential, challenges remain in addressing the toxicity associated with higher-generation dendrimers and in optimizing the drug loading and release kinetics, both of which require careful consideration during formulation development [29]. Recent reviews have highlighted the versatility of dendrimers as anticancer delivery platforms, emphasizing their capacity for multifunctional modification to enhance the targeting specificity and therapeutic payload [31]. For instance, dendrimers have been investigated for their ability to co-deliver anticancer agents and imaging probes, thereby improving the diagnostic accuracy while minimizing toxicity to healthy tissues [32].

Micelles, typically formed through the self-assembly of amphiphilic block copolymers in aqueous environments, represent another class of nanocarriers [10]. These core–shell structures, generally under 100 nm in diameter, contain a hydrophobic core suitable for encapsulating non-polar compounds and a hydrophilic shell that interfaces with the biological environment, improving their stability and circulation time [10,27]. Although native melittin is amphipathic and water-soluble, several strategies have been explored to enhance delivery, including complexation, conjugation, and incorporation into custom-designed polymeric systems [27,33]. For example, polyion complex micelles functionalized with targeting ligands such as estrone have been developed to enable the selective delivery of melittin to hormone-responsive breast cancer cells [33]. Stimuli-responsive micelles, which release their payload in response to specific triggers within the TME—such as a low pH or tumor-associated enzymes—also show considerable promise [27].

Inorganic nanoparticles—such as gold nanoparticles, mesoporous silica nanoparticles, and carbon nanotubes—offer robust platforms with distinctive physicochemical properties [10]. Their rigid structures provide high stability, and their surfaces can easily be functionalized to enable melittin conjugation [10]. Gold nanoparticles, for example, support straightforward surface modification through thiol linkages and have been functionalized with aptamers to achieve targeted melittin delivery to breast cancer cells [34]. Mesoporous silica nanoparticles, known for their large surface areas and tunable pore sizes, serve as versatile carriers for various therapeutic biomolecules, including peptides, allowing for substantial loading and controlled release, often triggered by pH changes or other environmental stimuli [10,35].

The selection of an optimal nanocarrier depends on the specific therapeutic objective, cancer type, and desired release characteristics. The current research continues to refine each platform to maximize the effectiveness of melittin-based nanotherapy in oncology [10,11].

### 3.2. Strategies for Enhanced Targeting and Delivery

Although nanocarriers provide passive targeting advantages according to the enhanced permeability and retention effect characteristic of tumor tissues, further enhancements in melittin’s delivery and release at the tumor site remain essential for improving its therapeutic efficacy and minimizing its systemic toxicity [10,34,36]. To achieve this level of precision, several active targeting and stimuli-responsive strategies have been developed, aiming to increase melittin’s accumulation and localized activity within tumor tissue [37].

One widely used strategy is ligand functionalization, in which the nanoparticle’s surface is modified with targeting moieties that bind to receptors or antigens overexpressed on cancer cells or tumor vasculature [37]. This form of active targeting enhances selective uptake and localization. Examples include antibodies or antibody fragments, such as anti-HER2 trastuzumab used in immunoliposomes [10]; peptides, including arginine–glycine–aspartic acid (RGD) peptides targeting αvβ3 integrins [10,36], chlorotoxin targeting MMP-2-positive cells for gene delivery [38], and EGF [10]; aptamers such as AS1411, which targets nucleolin [34]; small molecules like hyaluronic acid (HA), which targets CD44 [26]; and hormones such as estrone, which targets estrogen-receptor-positive cells [33]. Ligand selection is guided by the molecular expression profile of the specific cancer type [37].

Targeting the unique conditions of the TME or intracellular compartments offers an additional strategy for enhancing delivery through stimuli-responsive systems [39]. In addition to pH sensitivity—triggered by the acidic conditions of the TME or the endo/lysosomal compartments through the use of pH-responsive polymers or linkers [27,40]—other internal triggers have also been explored. Redox-responsive systems take advantage of the elevated glutathione (GSH) concentrations found inside cancer cells relative to those in the bloodstream. Nanocarriers incorporating disulfide bonds can remain stable in circulation but undergo cleavage in the reductive intracellular environment, releasing melittin at the target site [41]. Enzyme-responsive strategies have also been developed; for instance, melittin prodrugs can be engineered to remain inactive until cleaved by specific enzymes overexpressed in the TME, such as MMPs, and are typically delivered via nanoparticle carriers [42]. Although less frequently applied specifically to melittin-based systems, temperature- and light-responsive platforms represent additional stimuli-responsive strategies under investigation in the broader field of nanomedicine [39].

Prolonged circulation is essential for effective targeting, making immune evasion strategies a critical aspect of nanoparticle design [43]. Surface modification with PEG (PEGylation) remains the most widely used method of forming a hydrophilic barrier, reducing opsonization, limiting the uptake by the mononuclear phagocyte system, and extending the circulation time [28,43]. However, the immunogenicity of PEG—resulting in the formation of anti-PEG antibodies and the accelerated blood clearance phenomenon upon repeated administration—has emerged as a significant obstacle. This challenge has been observed even in preclinical studies involving melittin-loaded PEGylated micelles [27,44]. To overcome this limitation, several alternative strategies have been investigated. One approach involves chemical modification of the therapeutic payload itself; for example, substituting L-amino acids with D-amino acid isomers of melittin has been shown to significantly reduce immunogenicity while preserving cytolytic activity [27]. Other strategies have involved exploring the use of alternative stealth polymers, such as zwitterionic polymers, or biomimetic coatings using materials like cell membranes or CD47-based “don’t eat me” signals. These methods aim to prolong the circulation time while minimizing immune recognition [45]. Although specific applications to melittin-based nanoparticles remain limited, these polymers offer promising potential as PEG alternatives. Collectively, these diverse strategies—ligand functionalization, multi-stimuli responsiveness, and advanced immune evasion strategies—continue to evolve as key approaches to improving the therapeutic index of melittin nanotherapies [10,37].

### 3.3. Challenges in Melittin Nanomedicine

As discussed earlier, multiple strategies have been investigated for mitigating the toxicity profile of melittin, a principal component of bee venom. Melittin exerts direct cytotoxic effects on mammalian cells by inducing cytolysis through several mechanisms. These include increasing the water permeability of the cellular membrane, reducing membrane surface tension, and disrupting membrane charge balance. These effects are facilitated by the positive charge of melittin relative to the negatively charged eukaryotic cell membrane, ultimately resulting in pore formation. Intramuscular administration of melittin has also been associated with localized toxicity, characterized by hypercontractility of the myofibrils and subsequent muscle necrosis [46]. In addition to its cytolytic activity, melittin exhibits genotoxic potential. It promotes oxidative stress by increasing ROS levels and reducing GSH concentrations, which may lead to DNA strand breaks [47].

Following its systemic administration, regardless of the delivery route, melittin enters the bloodstream to exert anti-neoplastic effects. However, it exhibits pronounced toxicity toward human red blood cells due to its strong hemolytic activity. Blondelle et al. demonstrated that structural modifications of melittin can significantly influence its hemolytic potency, highlighting the role of specific amino acid residues in mediating membrane disruption [48]. In addition to its hemolytic effects, melittin has been associated with the disruption of coagulation homeostasis. It can induce both thrombotic and thrombolytic responses—either by activating platelets or promoting fibrinolysis—resulting in a range of adverse outcomes, including thrombosis and hemorrhage [49,50].

**Figure 4:**
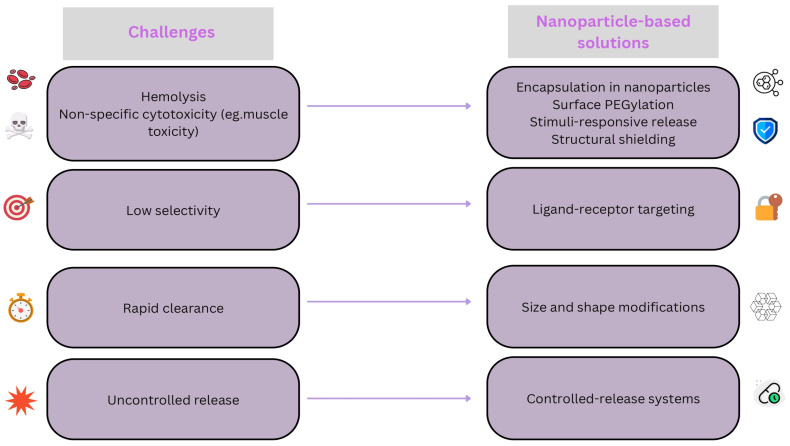
Challenges of melittin nanotherapy and corresponding nanoparticle-based solutions.

Similar to other components of bee venom, melittin functions as a foreign antigen and serves as a potential allergen capable of provoking life-threatening anaphylactic reactions. This immune response is primarily mediated through immunoglobulin E-dependent hypersensitivity pathways, ranging from localized inflammation to systemic anaphylaxis [49]. The reproductive system is also vulnerable to melittin-induced toxicity. Jallouk et al. reported dose-dependent cytotoxic effects on sperm cells and the vaginal epithelium, including reductions of 50% or more in sperm motility and viability, with progression to complete cellular elimination at higher concentrations [51]. These challenges have motivated the development of targeted nanoparticle strategies to enhance the safety and efficacy of melittin (Figure 4).

Given the severity of these adverse effects, extensive efforts have been dedicated to developing delivery systems that retain melittin’s antitumor efficacy while reducing its toxicity. Among these strategies, melittin-loaded nanoparticles have shown considerable promise. The initial approaches focused on structural modification of the tryptophan residues in melittin, either through metal coordination (e.g., with ruthenium) or substitution with fluorescent molecules. Although these modifications successfully reduced its hemolytic activity, they also resulted in diminished anticancer potency [52,53].

Nanoparticle conjugation has subsequently gained attention as a strategy for tumor-specific delivery of melittin, incorporating both passive and active targeting mechanisms. Passive targeting primarily takes advantage of the enhanced permeability and retention effect. Owing to the leaky vasculature of tumors and impaired lymphatic drainage, nanoparticles coated with PEG can evade immune recognition and accumulate within tumor tissue [54,55]. Nanoparticles smaller than 100 nm are especially well suited to tumor penetration and lymphatic targeting, making them appropriate for cancers involving lymph node dissemination [56]. Tumor cells can internalize these nanoparticles through various endocytic pathways, including clathrin-mediated, caveolin-mediated, micropinocytosis, and clathrin/caveolin-independent mechanisms [57]. Structural modifications—such as non-spherical curvatures and bottlebrush PEG architectures—have been shown to extend the circulation time and reduce the clearance by phagocytic cells. Encapsulation of melittin within these nanostructures prevents direct hemolytic interactions with erythrocytes, thereby enhancing its overall biocompatibility [54,55].

Melittin-conjugated nanoparticles can be designed to enable controlled release of their therapeutic payload. For instance, platforms incorporating GSH-sensitive linkages have demonstrated the ability to disassemble in tumor environments with elevated GSH levels, allowing for triggered release within 48 h [58]. Applying this approach to melittin delivery may enhance time-dependent efficacy while reducing concentration-related toxicity. Another strategy involves incorporating melittin into lipid-based nanoparticles, including systems that mimic high-density lipoproteins. Embedding positively charged melittin within a neutral phospholipid bilayer can effectively shield its charge, thereby reducing its hemolytic activity and improving its safety [59].

In addition to passive targeting, active targeting strategies are under development for improving the specificity of melittin delivery. One approach involves fusion proteins that link melittin to targeting ligands such as gelonin, VEGF165, or disintegrin, which can bind to tumor-specific receptors including HER2 and receptors expressed in triple-negative breast cancer (TNBC) [18,60,61,62]. Aptamers—short DNA or RNA sequences that bind to specific extracellular matrix proteins like nucleolin—have also been employed to direct melittin toward malignant cells [63]. Furthermore, calcium carbonate nanoparticles have been investigated for their ability to selectively interact with cancer cells overexpressing mucin-1, facilitating targeted melittin delivery [64]. Additional ligand–receptor targeting strategies utilize molecules such as hyaluronic acid, chlorin e6, and folic acid to bind receptors that are overexpressed on tumor cells, thereby enhancing the specificity and safety of delivery [65,66,67]. Enzyme-responsive delivery systems are also being explored. These platforms exploit cancer-associated enzymes—whether secreted or membrane-bound—as biological triggers to cleave specific linkers or disrupt nanoparticle structures, allowing for selective drug release at the tumor site [68]. This approach offers promising potential for improving melittin targeting while reducing systemic toxicity.

The current quality control measures and manufacturing technologies for nanoparticle systems remain insufficiently developed. These gaps can lead to heterogeneous therapeutic responses and inconsistent outcomes, emphasizing the need for stringent regulatory oversight. Most of the research in cancer nanotechnology remains in the experimental phase, with limited progress toward preclinical or clinical application. As such, the implementation of rigorous regulatory frameworks and standardized manufacturing protocols is essential. These measures are critical to ensure the safety, reproducibility, and scalability of nanoparticle-based therapies as they advance toward clinical use. Without adequate oversight, the translation of nanoparticle technologies into the clinic may be delayed or compromised, potentially affecting both therapeutic efficacy and patient safety.

## 4. Applications of Melittin-Based Nanoparticles in Cancer Therapy

### 4.1. A Summary of the Studies Across Cancer Types

The potential of melittin as a broad-spectrum anticancer agent has led to extensive preclinical research focused on nanoparticle-based delivery systems designed to harness its potent cytotoxic effects while minimizing its systemic toxicity. Across a wide range of cancer models and nanocarrier platforms, studies consistently demonstrate the efficacy of melittin nanotherapy in both in vitro and in vivo settings. Reported mechanisms include direct membrane lysis, induction of apoptosis, and modulation of oncogenic signaling pathways [10,11,46]. Beyond direct cytotoxicity, melittin-loaded nanomedicines have also shown significant immunomodulatory effects. These include the induction of immunogenic cell death (ICD), enhanced maturation of antigen-presenting cells, increased infiltration and cytotoxic activity of the effector lymphocytes, and reprogramming of tumor-associated macrophages (TAMs) from an immunosuppressive to a pro-inflammatory phenotype. Together, these effects contribute to a more robust antitumor immune response [69]. Although melittin demonstrates potent cytotoxic effects against a wide range of cancer cells, its dose-dependent toxicity toward normal cells remains a major limitation. The toxicity threshold varies substantially depending on factors such as the cell type, delivery method, nanoparticle formulation, and exposure duration. For instance, some studies report half-maximal inhibitory concentration (IC_50_) values in the low micromolar range for cancer cells, whereas normal cells may exhibit sensitivity at similar or only slightly higher concentrations. This narrow therapeutic window highlights the need for precise delivery systems that enhance tumor selectivity while minimizing systemic toxicity.

#### 4.1.1. Breast Cancer

Research on melittin-loaded nanoparticles for breast cancer is particularly extensive, reflecting the high prevalence and heterogeneity of the disease. Targeted delivery strategies have shown significant promise. For example, gold nanoparticles functionalized with both melittin and the AS1411 aptamer exhibited enhanced cytotoxicity against nucleolin-expressing MCF-7 breast cancer cells, with reduced effects on non-target cells [34]. Similarly, estrone-decorated polyion complex micelles were developed to deliver melittin specifically to hormone-responsive (estrogen-receptor-positive) breast cancer cells, improving the targeting specificity [33].

In the case of TNBC, a subtype often resistant to the standard therapies, nanoparticles delivering D-melittin via pH-sensitive polymers effectively suppressed the tumor growth in 4T1 mouse models and exhibited lower immunogenicity than that of L-melittin formulations [27]. An optimized variant, PEG-melittin-dKLA 8–26, was also developed to enhance its stability and reduce its hemolytic toxicity. In 4T1 TNBC models, this formulation significantly inhibited tumor growth, extended survival, and suppressed lung metastases by selectively targeting M2-like TAMs and modulating the TME [70].

Lipid-coated polymeric nanoparticles carrying melittin demonstrated significant tumor suppression in 4T1 models with minimal systemic toxicity [28]. Similarly, melittin-loaded niosomes showed superior cytotoxicity, inhibition of migration and invasion, and tumor growth reductions in both 4T1 and SKBR3 breast cancer models compared to these properties with free melittin, supporting the potential of niosomes as an effective delivery platform [71]. Studies using carbon-based carriers, such as graphene or graphene oxide, reported enhanced cytotoxic effects on both MDA-MB-231 (TNBC) and MCF-7 cells relative to those of free melittin [72,73].

Mechanistic investigations suggest that melittin suppresses breast cancer progression by inhibiting growth factor receptor activation (e.g., HER2) and inducing apoptosis through caspase activation and modulation of the Bax/Bcl-2 pathways [11,74]. Notably, the dual secured nano-sting formulation of melittin—a redox- and pH-responsive zwitterionic polymer complex—demonstrated substantial cytotoxicity against MCF-7 breast cancer cells in vitro while preserving red blood cell integrity, highlighting its promise for safe systemic delivery [41]. Furthermore, melittin can inhibit the signaling pathways associated with multidrug resistance (MDR), including PI3K/Akt and NF-κB, which regulate P-glycoprotein (P-gp) expression. Building on this, polymersomes based on poly(lactic acid)–hyaluronic acid (PLA-HA) have been designed to co-deliver melittin and doxorubicin, aiming to overcome MDR and enhance chemotherapeutic efficacy through combined cytotoxic and resistance-modulating mechanisms [73]. While common targeting ligands such as folic acid are frequently used in breast cancer nanomedicine, specific applications involving melittin-loaded nanoparticles remain limited in recent reports. Nonetheless, the general principle of ligand-mediated targeting remains highly applicable in this context [37].

#### 4.1.2. Melanoma

Melittin-loaded nanoparticles have demonstrated considerable efficacy against melanoma. Hyaluronic-acid-coated liposomes encapsulating melittin showed enhanced cellular uptake and cytotoxicity in CD44-positive B16F10 melanoma cells in vitro, suggesting the potential for active targeting-based therapeutic strategies [26]. A pivotal early study employed perfluorocarbon nanoparticles functionalized with αvβ3 integrin-targeting peptides to deliver melittin selectively to the tumor vasculature and melanoma cells in mice. This approach significantly inhibited tumor growth and induced apoptosis without detectable toxicity [36]. Another innovative strategy utilized hybrid α-melittin peptides engineered to self-assemble into ultrasmall lipid nanoparticles (~20 nm). Intravenous administration of these nanoparticles in B16F10 melanoma-bearing mice led to 82.8% tumor growth inhibition with no observable side effects, highlighting the potential of structurally modified melittin within a nanoplatform [59].

Complementary in vitro studies using free melittin have confirmed its potent activity against multiple melanoma cell lines, including human A375SM and SK-MEL-28 cells. These investigations demonstrated the suppression of proliferation, clonogenic survival, migration, and invasion, along with apoptosis induction through caspase-3 and caspase-9 activation. Mechanistically, melittin was found to downregulate key survival pathways, including the PI3K/AKT/mTOR and MAPK signaling cascades [75]. Although recent studies combining melittin nanoparticles with melanoma immunotherapies or BRAF inhibitors remain limited, the demonstrated effectiveness of melittin nanocarriers supports future exploration of combination strategies.

#### 4.1.3. Hepatocellular Carcinoma (HCC)

Liver cancer remains a significant global health concern. Notable preclinical success has been reported using melittin-loaded nanoliposomes stabilized with poloxamer 188 [76]. This formulation effectively inhibited HCC cell survival in vitro, including in SMMC-7721 cells, and suppressed tumor growth in both subcutaneous and orthotopic HCC mouse models in vivo. Importantly, these nanoliposomes caused substantially less inflammation and reduced allergic responses than those using free melittin, indicating improved biocompatibility and a potentially broader therapeutic window [76].

Mechanistic studies in HCC cell lines suggest that melittin acts through multiple pathways, including the inhibition of cell cycle progression—specifically G0/G1 arrest via modulation of the methyl-CpG-binding protein 2/Sonic hedgehog signaling axis—disruption of survival signaling, such as the histone deacetylase 2/phosphatase and tensin homolog/Akt pathway, and the induction of apoptosis [10]. Furthermore, melittin has been shown to sensitize HCC cells to tumor necrosis factor-related apoptosis-inducing ligand (TRAIL)-mediated apoptosis, indicating its potential for use in combination regimens, especially for TRAIL-resistant tumors [10]. While the poloxamer-stabilized nanoliposome formulation offers a strong proof of concept, further studies investigating alternative nanocarriers and targeted delivery strategies for melittin in HCC are warranted.

#### 4.1.4. Ovarian Cancer

Melittin has shown considerable potential against ovarian cancer cells in vitro, primarily by inducing apoptosis and modulating key signaling pathways. Studies using ovarian cancer cell lines like SKOV3 and PA-1 reported that free melittin treatment upregulates death receptors (DR3, DR4, DR6), activates caspase-3 and caspase-8, and inhibits the pro-survival Janus kinase 2 (JAK2)/STAT3 pathway [77]. More recently, melittin has also been identified as a modulator of the lipid metabolism in ovarian cancer cells, where it inhibits the nuclear translocation of sterol regulatory element-binding protein 1 (SREBP1) and reduces the SREBP1-driven transcription of fatty acid synthase, thereby suppressing lipogenesis and cell growth [78].

Building on this cytotoxic potential, nanoparticle-based strategies are now emerging. One recent study developed silica–alginate–melittin nanoconjugates (SAMNs) designed for controlled release through alginate lyase activation [79]. In vitro experiments using SKOV3 cells demonstrated that the SAMNs effectively inhibited cell proliferation, migration, and invasion while providing more sustained control than that with free melittin; the release mechanism also helped mitigate mitochondrial dysfunction [79]. Moreover, the dual secured nano-sting melittin platform effectively killed both SKOV3 and drug-resistant NCI/ADR-RES ovarian cancer cells in vitro, supporting its potential utility in overcoming chemoresistance while minimizing hemolytic toxicity [41]. In addition, p5RHH nanoparticles—derived from melittin—have been successfully employed to deliver AXL-targeting small interfering RNA (siRNA), significantly reducing the invasion, migration, and metastasis of ovarian cancer cells in both in vitro and in vivo models [80].

The potential for combination therapy has also been investigated. In vitro metabolomic studies combining free melittin with cisplatin—a standard chemotherapeutic agent for ovarian cancer—revealed synergistic effects in both cisplatin-sensitive (A2780) and cisplatin-resistant (A2780CR) ovarian cancer cell lines [81]. This combination significantly disrupted key metabolic pathways, including the tricarboxylic acid cycle and nucleotide metabolism, suggesting that nanoparticle-based co-delivery could enhance cisplatin’s efficacy or help overcome resistance [81]. Although nanoparticle strategies specifically targeting ovarian cancer—such as using ligands for folate receptor alpha, which is frequently overexpressed in this malignancy—remain underexplored [37], recent findings provide a promising foundation for the development of melittin-based nanotherapies. Further in vivo studies employing these or related nanoparticle formulations are essential to advance its clinical translation.

#### 4.1.5. Colorectal Cancer

Preclinical evidence supports the use of melittin-based nanotherapy for CRC, a malignancy that remains particularly difficult to manage in advanced or metastatic stages. Several nanoparticle formulations have shown promising results. D-melittin loaded into pH-sensitive polymeric micelles demonstrated significant antitumor efficacy in the CT26 syngeneic mouse colon carcinoma model [27]. Another strategy utilized redox-sensitive zwitterionic glycol chitosan complexes to deliver melittin, resulting in potent cytotoxicity against HCT-116 colon cancer cells in vitro with minimal hemolytic toxicity. These systems exploit the reductive intracellular environment for controlled release [41].

Beyond these delivery platforms, recent studies examining the activity of free melittin have revealed key anti-CRC mechanisms that extend beyond direct cytotoxicity. Melittin induces mitochondrial-pathway-mediated apoptosis by generating ROS, reducing the mitochondrial membrane potential, regulating the expression of Bax/Bcl-2, and promoting cytochrome c release [82]. Importantly, melittin also exhibits strong anti-metastatic activity. In vitro, it inhibits the migration and invasion of CRC cells, and in vivo, it suppresses lung metastasis development. These effects have been linked to the downregulation of MMPs and suppression of the EMT [82]. Although recent studies did not prominently feature melittin nanoparticle systems co-delivered with standard CRC chemotherapeutics such as 5-fluorouracil or oxaliplatin, nor did they include targeting ligands against markers like EGFR [83], the demonstrated efficacy of melittin nanocarriers [27,41] and the potent anti-metastatic mechanisms of melittin [82] strongly support the continued development of advanced nanoparticle strategies for CRC treatment.

#### 4.1.6. Prostate Cancer

Prostate cancer, given its typically long latency, represents a viable target for therapeutic intervention. Preclinical work, primarily reported in a 2018 conference abstract, introduced a formulation termed “NanoBees” which employed perfluorocarbon nanoemulsions to deliver melittin [84]. This study demonstrated reduced cell viability and apoptosis induction in vitro across several prostate cancer cell lines (DU-145, C42, PC3), with the underlying mechanisms involving the modulation of Bax/Bcl-2 expression, poly(ADP-ribose) polymerase cleavage, caspase activation, and inhibition of the PI3K/Akt signaling pathway. In vivo, administration of this formulation reportedly reduced the tumor volume in PC3 xenograft models and avoided the hemolytic toxicity associated with native melittin [84].

Alternative nanostrategies have also been explored. One gene therapy approach utilized chlorotoxin-targeted polyethylenimine nanoparticles to deliver a melittin-encoding gene to MMP-2-positive PC3 cells, resulting in reduced cell viability in vitro [38]. Although targeting ligands such as those against prostate-specific membrane antigen (PSMA) are commonly used in prostate cancer nanomedicine, specific applications involving PSMA-targeted melittin-based nanotherapy were not identified in recent studies. Therefore, while the preliminary data indicate promising therapeutic potential, further research involving fully published studies, expanded nanoparticle platforms, and optimized targeting strategies is necessary to validate melittin nanotherapy for prostate cancer. Supporting this perspective, a recent review summarized the anticancer properties of melittin and other bee venom components in prostate cancer models, highlighting a range of explored targets, such as MMP-2, PSMA, fibroblast activation protein, and hormone receptors [85].

#### 4.1.7. Pancreatic Cancer

Pancreatic cancer is frequently diagnosed at an advanced stage and carries a poor prognosis, emphasizing the urgent need for novel therapeutic strategies. Melittin-loaded nanoparticles are being investigated primarily in combination approaches, with the studies so far focused on in vitro applications. One study developed melittin-functionalized lipidic nanovesicles co-loaded with raloxifene, a selective estrogen receptor modulator [86]. In experiments using PANC1 pancreatic cancer cells, this nanoformulation significantly lowered the IC_50_ value compared to that with free raloxifene, induced G2–M phase cell cycle arrest, and enhanced apoptosis through the modulation of key signaling pathways, including Bax/Bcl-2 and TNF/NF-κB, indicating its synergistic anticancer potential [86].

Another study formulated icariin—a flavonoid glycoside—within melittin-decorated bilosomes. This system was optimized using an experimental design methodology and aimed to combine the cytotoxic effects of both agents for enhanced anticancer activity in vitro. The optimized formulation exhibited significantly enhanced anticancer activity, with an IC_50_ value markedly lower than that of free icariin and even lower than that of erlotinib, a standard therapeutic agent [87]. Despite these encouraging results, a critical limitation remains: searches of the recent literature did not reveal clearly documented studies evaluating the in vivo efficacy of melittin-based nanoparticles in pancreatic cancer models. Successful translation of these approaches will require overcoming major barriers, particularly the dense desmoplastic stroma characteristic of pancreatic tumors, which significantly impairs nanoparticle penetration and drug delivery [88]. Future research must prioritize the development of delivery platforms capable of navigating this restrictive TME and demonstrating efficacy in preclinical models in vivo.

#### 4.1.8. Lung Cancer

Melittin has shown promising potential against NSCLC, the most prevalent subtype of lung cancer, with growing interest in combination therapies and TME-modulating strategies. In vitro studies have demonstrated synergistic effects between free melittin and EGFR tyrosine kinase inhibitors (TKIs). One recent investigation reported that melittin enhanced the anticancer activity of erlotinib in A549 NSCLC cells by promoting apoptosis, potentially through interactions with JAK2/JAK3 kinases [89]. Another study compared melittin from different bee species and confirmed its synergistic cytotoxicity when combined with gefitinib against NSCLC cells in vitro [90]. However, both studies employed the free peptide rather than nanoparticle formulations.

A notable advancement toward nanoparticle-relevant in vivo applications involved the development of a hybrid peptide, melittin-dKLA, which self-assembles into nanofibers and exploits the affinity of melittin for M2 macrophages [91]. In a lung cancer mouse model, this construct successfully targeted M2-like TAMs and exerted anticancer effects, supporting the feasibility of TME-modulating strategies for melittin-based nanostructures [91]. Although EGFR is a well-established target for nanoparticle-based drug delivery in NSCLC [92], the recent literature did not reveal studies that specifically integrated EGFR-targeted nanoparticles with melittin delivery. Future research could focus on nanoparticle platforms capable of co-delivering melittin with TKIs or other agents, potentially combining active targeting with TME modulation, as exemplified by the melittin-dKLA approach.

#### 4.1.9. Glioblastoma

Glioblastoma represents a major therapeutic challenge due to its aggressive infiltration, inherent resistance to treatment, and the presence of the blood–brain barrier (BBB), which restricts drug penetration [93]. Although melittin exhibits potent cytotoxicity against glioblastoma cell lines in vitro, effective in vivo delivery via nanoparticles remains a significant obstacle. For instance, an in vitro study demonstrated that a bee venom fraction containing over 69% melittin completely reduced the viability of two different glioblastoma cell lines (LN18 and LN229) at concentrations as low as 2.5 to 3.5 µg/mL. This melittin-containing fraction also inhibited the activity of matrix metalloproteinases (MMP-2 and MMP-9) in a dose-dependent manner, suggesting a mechanism for reducing the invasive potential of this tumor. However, the authors note that these promising in vitro results must be validated in animal models, as parenteral administration of melittin is associated with high toxicity, and the challenge of delivering the compound across the BBB remains [94]. Another study confirmed a similar tumor-killing dose of 2.5 µg/mL for melittin, showing it acted synergistically with the chemotherapy drug cisplatin to further decrease cell viability and increase cell death in DBTRG-05MG glioblastoma cells. Mechanistically, melittin was shown to enhance the effects of cisplatin by activating the transient receptor potential cation channel, subfamily M, member 2 ion channel, which increased intracellular calcium and mitochondrial ROS, leading to greater apoptosis [95]. Despite this promising mechanistic evidence at the cellular level, direct evidence supporting the ability of melittin-loaded nanoparticles to cross the BBB and replicate these effects in vivo is limited, and the need for animal studies remains.

Recent reviews have also highlighted the use of nanoparticle-like delivery systems, such as targeted lipodisks, to deliver melittin in glioma models [10]. For example, lipodisks functionalized with c(RGDyK) peptides have been employed to target U87 glioma cells, while EGF-targeted lipodisks demonstrated superior cytotoxicity compared to that of free melittin [10]. Notably, targeted lipodisks have also been utilized to co-deliver melittin and the chemotherapeutic agent paclitaxel. This combination produced synergistic cytotoxic effects in vitro and reduced the tumor burden in intracranial glioma mouse models, supporting the potential of such systems for combination therapy in vivo [10]. These findings indicate that while conventional nanoparticles may be limited by the BBB, advanced targeted constructs such as lipodisks offer a promising platform for melittin-based therapies in glioblastoma. Further research is needed to optimize the delivery to the brain and validate efficacy in relevant in vivo models.

#### 4.1.10. Osteosarcoma

Recent research has focused on the development of advanced melittin-based nanocarriers for osteosarcoma, a primary bone malignancy that predominantly affects younger individuals. One promising strategy involves aptamer-modified melittin micelles, which were shown to effectively inhibit osteosarcoma progression in vivo by inducing ICD. One study confirmed the ICD effect within the TME of treated mice by showing key damage-associated molecular patterns, including the translocation of calreticulin to the cell surface and the extracellular release of high-mobility group box 1. This process successfully led to dendritic cell maturation and a significant increase in the infiltration of both CD4+ and CD8+ T cells into the tumor, confirming a potent, short-term antitumor immune response. However, while this study suggests the potential for “long-term benefits”, it did not include experiments such as a tumor rechallenge to demonstrate the formation of long-term immunological memory or the prevention of tumor recurrence [96]. Furthermore, detailed reports confirming melittin-induced ICD in vivo for other cancer types are still limited.

Melittin has also demonstrated direct cytotoxic effects against multiple osteosarcoma cell lines in vitro. In MG-63 cells, melittin significantly reduced cell viability and suppressed proliferation, migration, and invasion in both two-dimensional and three-dimensional models [97]. Furthermore, melittin has been shown to downregulate the expression of MMP-2 and MMP-9 in osteosarcoma cells, suggesting its potential anti-metastatic mechanism [98]. Together, these studies highlight the therapeutic potential of melittin nanocarriers for osteosarcoma by integrating targeted delivery, combination chemotherapy, ICD, and resistance-modulating effects. Continued exploration of these strategies may lead to more effective treatment options for this challenging cancer.

#### 4.1.11. Leukemia and Lymphoma

Melittin has exhibited cytotoxic activity in vitro against various leukemia and lymphoma cell lines, including U937 (histiocytic lymphoma/monoblastic leukemia), K562 (chronic myelogenous leukemia), CCRF-CEM (acute lymphoblastic leukemia), and KM-H2 and L-428 (Hodgkin lymphoma) [46,99]. The underlying mechanisms involve the induction of apoptosis, potentially via the intrinsic mitochondrial pathway and the modulation of signaling axes such as NF-κB/MAPK14 [46]. In addition, free melittin has been shown to sensitize Hodgkin lymphoma cells to cisplatin in vitro, suggesting its potential applications in combination therapy [99].

Despite this promise, translating melittin into an effective nanotherapeutic agent for hematological malignancies presents significant challenges, particularly in targeting circulating malignant cells while minimizing systemic toxicity. Although nanoparticle strategies targeting leukemia or lymphoma-specific markers such as CD19 and CD20 have been developed for other agents [100] and melittin-derived peptides have been incorporated into nanocomplexes for siRNA delivery in vitro [101], dedicated studies evaluating melittin-loaded formulations—such as liposomes or polymeric nanoparticles—for systemic delivery in leukemia or lymphoma models in vivo remain notably scarce in the recent literature. Further investigation is necessary to design safe and effective nanoparticle systems capable of delivering melittin for the treatment of these hematological cancers.

#### 4.1.12. Gastric Cancer

Preclinical studies highlight the therapeutic potential of melittin in gastric cancer. In vitro, purified melittin has been shown to inhibit the proliferation of human gastric adenocarcinoma AGS cells in a dose- and time-dependent manner [102]. Furthermore, melittin exhibits notable anti-metastatic effects at lower concentrations by reducing cell adhesion, colony formation, migration, and invasion. These effects may involve suppression of the EMT through modulation of the Wnt/BMP signaling pathways [22]. Apoptosis induction has also been documented in other gastric cancer cell lines, such as SGC-7901 [22]. Despite these encouraging in vitro findings with free melittin, studies investigating nanoparticle-mediated delivery to enhance efficacy or enable targeted therapy in gastric cancer models remain limited. Further research is warranted to explore nanocarrier strategies tailored to this malignancy.

#### 4.1.13. Cervical Cancer

Melittin has shown direct cytotoxic effects on cervical cancer cells in vitro. In a study using purified melittin, treatment of HeLa human cervical cancer cells resulted in inhibited proliferation and increased apoptosis, as demonstrated through the MTT assay and flow cytometry [13]. Although these findings support the potential utility of melittin for cervical cancer therapy, studies employing specific nanoparticle delivery systems to enhance melittin’s efficacy or targeting in this context were not identified in the recent literature.

#### 4.1.14. Bladder Cancer

Melittin has demonstrated cytotoxic activity against bladder cancer cells in vitro. Purified melittin inhibited the proliferation of human bladder cancer cells in a dose-dependent manner and induced apoptosis through a mechanism involving the inhibition of microRNA-146a-5p maturation via methyltransferase-like 3 targeting, which in turn activated the NUMB/NOTCH2 signaling pathway [103]. In vivo, intratumoral administration of melittin significantly reduced the tumor growth in bladder cancer xenograft mouse models [103]. These preclinical findings highlight the therapeutic potential of melittin in bladder cancer; however, further investigation is needed to develop systemic delivery strategies, such as nanoparticle-based platforms, that can enhance the targeting specificity and facilitate clinical translation.

#### 4.1.15. Thyroid Cancer

Nanoparticle-based strategies have been explored for delivering bee venom components, including melittin, in thyroid cancer models. One study investigated zeolitic imidazolate framework-8 metal–organic framework nanoparticles loaded with bee venom in vitro using TT medullary thyroid cancer cells [104]. These nanoparticles exhibited cytotoxic activity, with IC_50_ values around 16–17 µg/mL. Treatment altered the expression of apoptosis- and cell-cycle-related genes, increasing pro-apoptotic Bax and caspase-3, while downregulating anti-apoptotic Bcl-2 and the cell cycle regulators cyclin D1 and cyclin-dependent kinase 4 [104]. This study supports the feasibility of metal–organic-framework-based nanoparticles as carriers for bee venom and melittin in thyroid cancer treatment strategies.

#### 4.1.16. Head and Neck Cancer

The potential of melittin in combination therapy for HNSCC has been explored, particularly in the context of radiosensitization. An in vitro study demonstrated that treatment with free melittin enhanced the radiosensitivity of hypoxic HNSCC cell lines (CNE-2, KB) [24]. This effect was linked to the ability of melittin to suppress HIF-1α, a key regulator of cellular adaptation to low-oxygen conditions [24]. Although this study did not incorporate nanoparticle-based delivery, its findings support further investigation into melittin nanocarriers as potential radiosensitizers for improving the radiotherapy outcomes in HNSCC.

#### 4.1.17. Esophageal Cancer

Similar to the findings in HNSCC, melittin has been investigated as a potential adjuvant to radiotherapy for esophageal squamous cell carcinoma (ESCC). Evidence from an in vivo ESCC xenograft mouse model indicates that melittin treatment enhances the therapeutic response to radiation [105]. Further studies incorporating nanoparticle-based delivery systems may help optimize this combination strategy.

#### 4.1.18. Endometrial Cancer

Although direct nanoparticle-based delivery of cytotoxic melittin for endometrial cancer has not been explored, melittin-derived components have been incorporated into nanomedicine platforms. One example involves a detoxified melittin-derived peptide, p5RHH, which was used in a nanoparticle system to deliver therapeutic AXL-targeting siRNA. This formulation demonstrated efficacy in inhibiting metastasis in preclinical models of uterine cancer [80]. These findings highlight the versatility of melittin-derived structures in the development of nanotherapeutic strategies.

#### 4.1.19. Other Cancers

Despite the broad-spectrum anticancer activity demonstrated by melittin, dedicated studies evaluating specific nanoparticle formulations against other malignancies such as non-melanoma skin cancers, renal cancers, and sarcoma subtypes beyond osteosarcoma were not prominently identified in the recent literature. These represent important areas for future investigation.

Overall, preclinical evidence supports the promise of melittin-based nanoparticles as a versatile anticancer strategy. Across numerous cancer types, nanoformulations have effectively delivered melittin; induced cancer cell death through mechanisms such as membrane lysis and apoptosis; inhibited tumor growth and metastasis in animal models; and improved the safety profiles compared to those of free melittin. A summary of selected preclinical studies employing melittin-based nanoparticles across various cancer types is provided in Table 1. Advancing this therapeutic approach will require continued research focused on optimizing active targeting, overcoming biological barriers such as the BBB, and conducting comprehensive long-term efficacy and safety studies. At present, no registered clinical trials specifically evaluating melittin nanoparticles for cancer therapy were identified, highlighting that this approach remains in the preclinical phase of development.

### 4.2. Its Role in Overcoming Drug Resistance

A major limitation to the long-term efficacy of cancer chemotherapy is the emergence of drug resistance, whether intrinsic or acquired, which results in reduced responsiveness of tumors to treatment [25]. Cancer cells utilize a variety of resistance mechanisms, including increased drug efflux, alterations in drug targets, the activation of pro-survival signaling pathways, and impairments in apoptotic processes [25,46]. Melittin, especially when delivered through nanoparticle-based systems, offers a multifaceted approach to overcoming these resistance pathways, providing a promising therapeutic option for treatment-refractory cancers [46].

One key advantage of melittin lies in its primary mechanism of action: direct disruption of the cell membrane, leading to cytolysis [10,46]. Unlike the conventional chemotherapeutics or targeted therapies that depend on specific intracellular pathways or molecular targets, melittin compromises membrane integrity by forming pores, inducing cell death largely independently of downstream processes [36,59]. This physical mode of action reduces the susceptibility to resistance mechanisms involving mutations in drug targets or impairments in apoptotic pathways [46]. Cancer cells are considered less likely to develop resistance to agents that cause physical membrane disruption [46].

Beyond direct cell lysis, melittin and its analogs have demonstrated the ability to interfere with specific resistance mechanisms. A key contributor to MDR is the overexpression of drug efflux pumps, particularly P-gp, which actively expels chemotherapeutic agents from cancer cells [25]. Melittin has been shown to modulate the signaling pathways involved in P-gp regulation, including PI3K/Akt and NF-κB [73]. In addition, a melittin analog (MEL-pep) directly inhibited P-gp expression and increased the intracellular accumulation of rhodamine-123, a P-gp substrate, in 5-fluorouracil-resistant HCC cells, an effect linked to the deactivation of the PI3K/Akt pathway [106]. Nanoparticle co-delivery systems, such as PLA-HA polymersomes encapsulating both melittin and doxorubicin, have been designed to exploit these properties in overcoming MDR [73]. Supporting their utility against resistant phenotypes, D-melittin-loaded polymeric nanoparticles retained strong cytotoxic effects in vitro against a doxorubicin-resistant breast cancer cell line [27]. Similarly, the redox- and pH-responsive dual secured nano-sting formulation of melittin exhibited significant cytotoxicity against the MDR ovarian cancer cell line NCI/ADR-RES, demonstrating its ability to bypass chemoresistance while minimizing hemolytic toxicity [41].

Melittin also functions as a chemosensitizer, enhancing the efficacy of conventional chemotherapeutic agents. Synergistic effects were observed in vitro when free melittin was combined with cisplatin in resistant ovarian cancer cells [81], as well as with EGFR inhibitors in NSCLC cells [89,90]. Nanoparticle-based delivery platforms are well suited to co-delivering melittin and standard chemotherapeutic drugs at optimized ratios, allowing for controlled release and maximizing synergistic interactions in vivo [25].

Emerging evidence suggests that melittin may also induce ICD [27,96]. By causing rapid cell lysis or inducing apoptosis, melittin can trigger the release of damage-associated molecular patterns and tumor-associated antigens, potentially activating an antitumor immune response [46]. This immunostimulatory effect may help eliminate residual or therapy-resistant cancer cells, further enhancing the therapeutic impact of melittin-based treatments.

Nanoparticle-based delivery plays a critical role in translating the resistance-overcoming properties of melittin into effective therapies. Nanocarriers shield melittin from enzymatic degradation, mitigate its systemic toxicity, allow for its controlled release, facilitate its co-delivery with conventional drugs, and offer the potential to selectively target resistant cancer cell populations [10,46].

Overall, melittin-based nanoparticles represent a promising strategy for overcoming drug resistance through multiple complementary mechanisms. These include bypassing the conventional resistance pathways via direct membrane disruption, downregulating drug efflux pump expression and activity, modulating resistance-associated signaling networks, sensitizing the cancer cells to chemotherapeutic agents, and inducing ICD. Although the in vitro data and mechanistic insights are compelling, further validation in well-characterized in vivo models of drug-resistant tumors is essential to support clinical translation.

### 4.3. Combination with Other Therapies

To improve therapeutic outcomes, address tumor heterogeneity, and overcome resistance, combination therapy has become a central strategy in modern cancer treatment. Melittin-based nanoparticles are particularly well suited to such approaches due to the broad spectrum of mechanisms employed by melittin and the flexibility of nanocarrier systems. These platforms enable the co-delivery of multiple agents or synergy with other treatment modalities, including chemotherapy, immunotherapy, photothermal therapy (PTT), and radiotherapy [10,46].

#### 4.3.1. Combination with Chemotherapy

One of the most actively investigated strategies involves combining melittin nanoparticles with conventional chemotherapeutic agents. Nanoparticle systems enable the co-encapsulation and simultaneous delivery of melittin and a cytotoxic drug, offering potential benefits such as synergistic effects, reduced dosing of toxic agents, and the ability to overcome MDR [25,46]. As discussed earlier, melittin inhibits drug efflux pumps like P-gp and enhances the sensitivity to chemotherapy [73,81,106]. Specific nanoparticle formulations have been engineered to harness these properties. For instance, PLA-HA polymersomes co-loaded with melittin and doxorubicin were developed to address MDR [73]. Similarly, targeted lipodisks co-delivering melittin and paclitaxel exhibited synergistic cytotoxicity in vitro and tumor suppression in in vivo glioma models [10]. Moreover, although many studies have employed free melittin in vitro, the observed synergies with EGFR inhibitors such as erlotinib and gefitinib in NSCLC cells [89,90] and with cisplatin in resistant ovarian cancer cells [81] support the rationale for developing nanoparticle-based co-delivery systems for these combinations. In addition to nanoparticle-based co-delivery systems, melittin has been incorporated into fusion proteins including VEGF–melittin, ATF–melittin, and melittin–interleukin-2. These constructs are designed to target the tumor vasculature or immune checkpoints while minimizing the toxicity to healthy tissues and have demonstrated selective antitumor effects in preclinical models of ovarian, liver, and prostate cancer [107].

#### 4.3.2. Combination with Immunotherapy

Melittin interacts with the immune system in ways that offer promising opportunities for its combination with immunotherapy. It can induce ICD [27,96], potentially priming an adaptive immune response. Nanoparticle strategies enable further modulation of the TME to enhance antitumor immunity. One notable example involves manganese dioxide (MnO_2_)–melittin nanoparticles, where the MnO_2_ component alleviates tumor hypoxia by reacting with endogenous hydrogen peroxide, thereby reducing immunosuppressive factors such as programmed death-ligand 1 upregulation [108]. In combination with melittin-mediated cytotoxicity, these nanoparticles enhanced systemic antitumor immune responses in vivo [108]. Additional studies have shown that melittin synergizes with phospholipase A_2_ derived from bee venom, particularly in CRC models. This combination increased apoptosis in HCT116 cells and may enhance tumor antigen release to support immune priming [107]. Other approaches, such as self-assembling melittin-dKLA nanofibers targeting M2-like TAMs [91], also aim to reshape the TME. Although direct studies combining melittin nanoparticles with immune checkpoint inhibitors remain limited, the TME-modulating and ICD-inducing effects of melittin provide a strong rationale for pursuing such combinations in future research [46].

#### 4.3.3. Combination with Photothermal Therapy

PTT employs agents that convert near-infrared light into heat to ablate tumor cells. Combining PTT with melittin delivery through a single nanoparticle platform—for example, using gold nanoparticles [34] or other photothermal materials as carriers—offers a potential dual-pronged therapeutic strategy [10]. Hyperthermia may enhance melittin-induced cytotoxicity or trigger its release, while melittin contributes a direct membrane-disruptive mechanism. A related approach involves integrating melittin delivery with photodynamic therapy [10]. Studies demonstrating a combined chemo–photothermal effect using melittin-loaded nanoparticles were not prominently identified in the recent literature, highlighting this area as a target for future research. Nonetheless, several promising platforms have been developed that integrate melittin with photothermal- or redox-responsive agents such as chlorin e6, MnO_2_, and polyaniline. These systems exploit heat or ROS generation to induce melittin release while concurrently modulating the TME [10,107].

#### 4.3.4. Combination with Radiotherapy

Melittin has demonstrated potential as a radiosensitizer based on studies employing free melittin in vitro in hypoxic HNSCC cells [24] and in vivo in ESCC xenograft models [105]. Nanoparticle-based delivery may enhance the clinical applicability of this approach by concentrating melittin within the tumor during radiation exposure. However, specific studies evaluating melittin-loaded nanoparticles as radiosensitizers remain limited and warrant further investigation.

In summary, melittin-based nanoparticles hold considerable promise for incorporation into multimodal cancer treatment strategies. Their capacity for co-loading with chemotherapeutic agents, potential synergy with physical therapies such as PTT and radiotherapy, and ability to modulate the immune TME highlight their versatility. Future research should focus on optimizing these combination approaches, particularly by demonstrating in vivo efficacy, mechanistic synergy, and safety, to support the clinical translation of melittin nanotherapy.

## 5. Future Directions

While preclinical studies have established a strong foundation for melittin-based nanomedicine, significant barriers remain before its clinical implementation. Bridging the translational gap will require future research to prioritize advanced delivery optimization, thorough safety evaluations, and standardized manufacturing protocols. One key focus is the design of nanocarriers with improved biocompatibility and targeted delivery. Although PEGylation is commonly used to extend the circulation time, its associated immunogenicity has prompted investigations into alternatives such as biomimetic cell membrane coatings and advanced synthetic polymers like zwitterionic materials. These approaches aim to prolong its circulation while minimizing immune detection [45]. Furthermore, external stimuli-responsive nanoparticles—such as those activated by magnetic fields—may provide superior spatial and temporal control over melittin release compared to that in systems relying solely on TME cues. This enhanced precision could increase therapeutic efficacy and reduce off-target effects [109,110].

Rigorous preclinical and clinical validation remains essential for advancing melittin-based nanotherapy. Integrating diagnostic capabilities with therapeutic delivery—an approach known as theranostics—by incorporating imaging agents into melittin-loaded nanocarriers could enable non-invasive, real-time monitoring of its biodistribution, target engagement, and the treatment response, facilitating personalized adjustments [36]. Intratumoral heterogeneity, a persistent obstacle to targeted therapy [111], may be addressed through innovative solutions such as multi-targeted nanoparticles or combination strategies tailored to melittin delivery, although this area requires further exploration. Importantly, comprehensive studies assessing the long-term fate, potential tissue accumulation, and biodegradation products of nanocarriers are critical for understanding chronic toxicity beyond the immediate effects of melittin [112,113].

Manufacturing and regulatory considerations are paramount for successful clinical translation. Establishing robust, scalable manufacturing processes compliant with Good Manufacturing Practice (GMP) standards is essential to ensure batch-to-batch consistency in nanoparticle characteristics and therapeutic payload, which is necessary for reproducible outcomes and regulatory approval [114,115]. Clinical translation of melittin-based nanomedicines will require scalable production processes that meet the current GMP standards, including in terms of reproducibility, sterility, particle size control, and endotoxin limits. Regulatory agencies such as the U.S. Food and Drug Administration and the European Medicines Agency have published guidelines on the quality and safety requirements for nanoparticle drug products, including considerations on surface characterization, batch-to-batch consistency, and biological interactions [116]. Notably, several lipid- and polymer-based nanotherapeutics—such as Doxil and Abraxane—have successfully met the GMP requirements and advanced to regulatory approval, demonstrating the feasibility of translating complex nanosystems into the clinic [117,118]. These precedents offer a practical framework for the clinical development of melittin-loaded nanoparticles, provided that similar quality control standards and regulatory pathways are followed. To date, no melittin-based nanoparticle formulation has reached GMP-certified manufacturing or entered clinical trials, highlighting the need for future work to develop clinical-grade, scalable formulations that meet the regulatory standards on safety, efficacy, and reproducibility.

In parallel, the development and implementation of advanced computational tools—including physiologically based pharmacokinetic models and agent-based models—can support this process [119,120,121]. These computational and artificial-intelligence-driven models enable the prediction of human pharmacokinetics, optimization of the dosing strategies, and simulation of complex cellular and tissue-level interactions, thereby bridging the gap between the preclinical findings and clinical trial design [119,122]. However, while physiologically based pharmacokinetic and artificial-intelligence-driven models are increasingly used to optimize nanomedicine development in general, dedicated studies reporting their specific application to predicting the behavior and therapeutic efficacy of melittin-loaded nanoparticles are not yet prominent in the literature. Therefore, adapting and applying these advanced computational methods represent a critical and promising area for future research to accelerate the clinical translation of melittin nanotherapies. In addition, although combination therapies offer significant potential, optimizing their scheduling and dosing ratios for melittin nanoparticle administration remains essential [45,112]. Nanocarriers enable the co-delivery of multiple agents, but achieving the maximal therapeutic synergy will require rigorous preclinical evaluation, potentially supported by advanced modeling techniques and relevant ex vivo systems.

Future studies should focus on validating melittin-based nanocarriers in clinically relevant animal models, optimizing the dosing and delivery strategies, and evaluating therapeutic synergy with immunotherapies, chemotherapies, and radiotherapy. Parallel efforts are needed to develop scalable manufacturing techniques that comply with GMP standards and to characterize toxicity and pharmacokinetics comprehensively. In addition, integrating artificial intelligence and computational modeling may enhance the formulation design and predict the treatment response, accelerating progress toward clinical translation. Addressing these future directions through focused and collaborative research will be vital for overcoming the current limitations and ultimately advancing melittin-based anticancer nanomedicine toward safe and effective application in humans.

## 6. Conclusions

Melittin is a highly potent anticancer peptide with demonstrated mechanisms of action that include membrane disruption, the induction of apoptosis, and modulation of key signaling pathways. However, its clinical application is hindered by challenges such as hemolysis, immune activation, and unfavorable pharmacokinetics. Nanoparticle-based delivery systems have emerged as a promising approach, enabling targeted delivery, controlled release, and improved biocompatibility. These platforms are designed to harness both passive and active targeting strategies and have shown therapeutic efficacy across diverse cancer models.

Preclinical data support the use of melittin nanoparticles to enhance tumor specificity, reduce systemic toxicity, and overcome treatment resistance, particularly in combination with chemotherapy, radiotherapy, or immunotherapy. Despite this progress, clinical translation remains limited. Future efforts should prioritize optimization of the delivery strategies, the resolution of regulatory challenges, and rigorous in vivo validation. With continued advancements, melittin-based nanomedicine holds strong potential as a safe and effective cancer treatment.

## Figures and Tables

**Table 1:** Selected studies on melittin nanoparticle systems and key anticancer findings by cancer type.

Cancer Type	Nanoparticle System	Key Findings	Key Studies
Breast Cancer	Aptamer-functionalized gold nanoparticles	Enhanced targeting and cytotoxicity against MCF-7 cells	[34]
	D-melittin polymeric nanoparticles	Reduced immunogenicity and improved tumor suppression in TNBC models	[27]
	Melittin-loaded niosomes	Inhibited migration/invasion and reduced tumor growth in 4T1 and SKBR3 models	[71]
Melanoma	Hyaluronic-acid-coated liposomes	Enhanced uptake and cytotoxicity in CD44+ B16F10 melanoma cells	[26]
	Hybrid melittin lipid nanoparticles	82.8% tumor growth inhibition in B16F10 melanoma mice	[59]
Hepatocellular Carcinoma	Poloxamer-stabilized nanoliposomes	Suppressed tumor growth in vitro and in vivo; reduced inflammation and allergic responses	[76]
Ovarian Cancer	Silica–alginate–melittin nanoconjugates	Inhibited the proliferation, migration, and invasion of SKOV3 cells with sustained release	[79]
	p5RHH-siRNA nanoparticles	Suppressed invasion, migration, and metastasis in vitro and in vivo	[80]
Colorectal Cancer	Redox-sensitive glycol chitosan complexes	Induced apoptosis, suppressed EMT/metastasis via mitochondrial pathways and MMP downregulation	[82]
Prostate Cancer	Perfluorocarbon nanoemulsions	Induced apoptosis and inhibited the PI3K/Akt pathway; reduced the tumor volume without hemolysis	[84]
Pancreatic Cancer	Melittin-functionalized lipidic vesicles + raloxifene	Induced apoptosis and cell cycle arrest in PANC1 cells	[86]
Lung Cancer	Melittin-dKLA nanofibers	Targeted M2-like TAMs and reduced tumor growth in vivo	[91]
Glioblastoma	Lipodisks + c(RGDyK) + paclitaxel + melittin	Synergistic cytotoxicity and reduced tumor burden in intracranial models	[10]
Osteosarcoma	Aptamer-modified melittin micelles	Induced immunogenic cell death and inhibited tumor progression in vivo	[96]
Leukemia and Lymphoma	Free melittin	Induced apoptosis via the mitochondrial and NF-κB/MAPK14 pathways in multiple leukemia and lymphoma cell lines	[46,99]
Gastric Cancer	Purified melittin	Dose- and time-dependent inhibition oft he proliferation of AGS cells	[102]
Cervical Cancer	Purified melittin	Induced apoptosis and suppressed proliferation in HeLa cells	[13]
Bladder Cancer	Purified melittin	Suppressed proliferation via the miR-146a-5p/NUMB/NOTCH2 axis; effective reduction in tumor growth	[103]
Thyroid Cancer	Bee-venom-loaded ZIF-8 nanoparticles	Cytotoxic to TT cells via apoptosis-related gene modulation	[104]
Head and Neck Cancer	Free melittin	Enhanced radiosensitivity by suppressing HIF-1α in hypoxic HNSCC cells	[24]
Esophageal Cancer	Free melittin	Radiosensitization and apoptosis induction in vivo (ESCC models)	[105]
Endometrial Cancer	p5RHH-siRNA nanoparticles	Inhibited metastasis in uterine cancer models	[80]

## Data Availability

No new data were created or analyzed in this study. Data sharing does not apply to this article.

## Abbreviations

Akt	Protein kinase B
BBB	Blood–brain barrier
BMP	Bone morphogenetic protein
CRC	Colorectal cancer
DR	Death receptor
EGF	Epidermal growth factor
EGFR	Epidermal growth factor receptor
EMT	Epithelial–mesenchymal transition
ESCC	Esophageal squamous cell carcinoma
GMP	Good Manufacturing Practice
GSH	Glutathione
HA	Hyaluronic acid
HCC	Hepatocellular carcinoma
HER2	Human epidermal growth factor receptor 2
HIF-1α	Hypoxia-inducible factor 1-alpha
HNSCC	Head and neck squamous cell carcinoma
IC_50_	Half-maximal inhibitory concentration
ICD	Immunogenic cell death
JAK2	Janus kinase 2
MAPK	Mitogen-activated protein kinase
MDR	Multidrug resistance
MnO_2_	Manganese dioxide
MMP	Matrix metalloproteinase
mTOR	Mammalian target of rapamycin
MTT	3-(4,5-dimethylthiazol-2-yl)-2,5-diphenyltetrazolium bromide
NF-κB	Nuclear factor kappa B
NSCLC	Non-small-cell lung cancer
PEG	Polyethylene glycol
PI3K	Phosphoinositide 3-kinase
PLA-HA	Poly(lactic acid)–hyaluronic acid
P-gp	P-glycoprotein
PSMA	Prostate-specific membrane antigen
PTT	Photothermal therapy
RGD	Arginine–glycine–aspartic acid
ROS	Reactive oxygen species
SAMN	Silica–alginate–melittin nanoconjugate
siRNA	Small interfering RNA
SREBP1	Sterol regulatory element-binding protein 1
STAT3	Signal transducer and activator of transcription 3
TAM	Tumor-associated macrophage
TME	Tumor microenvironment
TNBC	Triple-negative breast cancer
TNF	Tumor necrosis factor
TRAIL	Tumor necrosis factor-related apoptosis-inducing ligand
TKIs	Tyrosine kinase inhibitors
VEGF	Vascular endothelial growth factor
